# Regular access and adherence to medications of the specialized component of pharmaceutical services

**DOI:** 10.11606/S1518-8787.2017051006932

**Published:** 2017-11-13

**Authors:** Janaína Soder Fritzen, Fabiane Raquel Motter, Vera Maria Vieira Paniz

**Affiliations:** I Universidade do Vale do Rio dos Sinos. Programa de Pós-Graduação em Saúde Coletiva. São Leopoldo, RS, Brasil

**Keywords:** Drug Utilization, Medication Adherence, Health Services Accessibility, Pharmaceutical Services, Pharmacoepidemiology, Uso de Medicamentos, Adesão à Medicação, Acesso aos Serviços de Saúde, Assistência Farmacêutica, Farmacoepidemiologia

## Abstract

**OBJECTIVE:**

To investigate the prevalence of the adherence to the medications of the Specialized Component of Pharmaceutical Services and its association with regular access in users of these medications in the municipality of São Leopoldo, State of Rio Grande do Sul, Brazil.

**METHODS:**

This is a cross-sectional study with adults aged 20 years and over, who are users of medications of the Specialized Component of Pharmaceutical Services. Sampling was carried out consecutively for users who accessed the service of the Specialized Component of Pharmaceutical Services during the period from December 2014 to March 2015. Adherence was measured by the Brief Medication Questionnaire, and adherents were defined as those who did not present barriers to adherence in the three domains. Regular access was defined as getting all medications in the last three months, regardless of how it was obtained (paying or for free). Data analysis was performed using Poisson regression with robust variance.

**RESULTS:**

We interviewed 414 subjects, being them mostly women (60.9%), with mean age of 55 years (SD = 13), and using a single medication of the Specialized Component of Pharmaceutical Services (68.1%). The prevalence of adherence to the medications used in the last seven days was 28.3% and the prevalence of free regular access was 46.1%, and 25.7% did not have access to all treatment. After adjusting for the number of medications in the Specialized Component of Pharmaceutical Services and the number of medications of continuous use, users who had free regular access in the last three months were 60% more likely to show adherence. For individuals with paid regular access, no association was found between access and adherence.

**CONCLUSIONS:**

The regularity in the free access to the medications of the Specialized Component of Pharmaceutical Services has an impact on the behavior of users, contributing to their commitment to treatment and self-care. The Specialized Component of Pharmaceutical Services needs programming in order to avoid irregular access, which suggests a significant limitation of the drug policies in Brazil.

## RESUMO

**OBJETIVO:**

Investigar a prevalência de adesão a medicamentos do Componente Especializado da Assistência Farmacêutica e sua associação com o acesso regular em usuários desses medicamentos no município de São Leopoldo, RS.

**MÉTODOS:**

Estudo transversal com adultos de 20 anos ou mais, usuários de medicamentos do Componente Especializado da Assistência Farmacêutica no município de São Leopoldo, RS. A amostragem foi realizada de forma consecutiva de usuários que acessaram o atendimento do Componente Especializado da Assistência Farmacêutica durante o período de dezembro de 2014 a março de 2015. A adesão foi mensurada pelo *Brief Medication Questionnaire*, sendo definidos como aderentes indivíduos que não apresentaram barreiras para a adesão nos três domínios. O acesso regular foi definido como a obtenção de todos os medicamentos nos últimos três meses, independentemente da forma de obtenção (pago ou gratuito). A análise dos dados foi realizada por regressão de Poisson com variância robusta.

**RESULTADOS:**

Foram entrevistados 414 indivíduos, a maioria mulheres (60,9%), média de idade de 55 anos (DP = 13), utilizando um único medicamento do Componente Especializado da Assistência Farmacêutica (68,1%). A prevalência de adesão aos medicamentos utilizados nos últimos sete dias foi de 28,3%, a prevalência de acesso regular gratuito foi de 46,1%; e 25,7% não tiveram acesso a todo o tratamento. Após ajuste para número de medicamentos do Componente Especializado da Assistência Farmacêutica e número de medicamentos de uso contínuo, usuários que tiveram acesso regular gratuito nos últimos três meses apresentaram 60% mais probabilidade de adesão. Para indivíduos com acesso regular mantido mediante pagamento, não foi encontrada associação entre o acesso e a adesão.

**CONCLUSÕES:**

A regularidade no acesso gratuito aos medicamentos do Componente Especializado da Assistência Farmacêutica tem impacto no comportamento do usuário, contribuindo para seu comprometimento com o tratamento e para o autocuidado. O Componente Especializado da Assistência Farmacêutica necessita de programação, a fim de evitar a irregularidade no acesso, que sugere uma importante limitação das políticas de medicamentos no país.

## INTRODUCTION

The effectiveness of the pharmacological treatment is closely related to the availability of the medication to the user, in an accessible way, as well as to the acceptance and commitment of the individual regarding treatment and rational use^a^.

In Brazil, the availability of medications in the Unified Health System (SUS) is organized into blocks of pharmaceutical funding, being the Specialized Component of Pharmaceutical Services (CEAF) the one dedicated to the acquisition and distribution of high-cost medications, which are established in Clinical Protocols and Therapeutic Guidelines (PCDT) and used at the outpatient level in the treatment of chronic and rare diseases. This block allocation defines those responsible for the funding in an attempt to ensure the regular provision of these medications to users^b,c^. Nonetheless, studies have shown that access to medication of continuous use, especially free of charge, is still limited: while access to all treatment covers approximately 85% of users, free access reach a little over half of them^12^ ^,^ ^13^.

Regarding the individual, studies have identified that adherence to essential medication ranges from 15.6% to 76.8%^5^ ^,^ ^7^ ^,^ ^8^, but the way in which the users of the CEAF use their medications, or the adherence to treatment, is still unknown. Adherence is defined by the World Health Organization as the degree to which the behavior of a person, represented by taking medication, following a diet, or changing the lifestyle, is in accordance with the recommendations of a health care professional.

In this sense, the regular access to medication and adherence of the individual to recommendations are essential for the rational use of medications, especially those of high cost – such as the medications of the CEAF. The objective of this study was to investigate the prevalence of the adherence to the medications of the Specialized Component of Pharmaceutical Services and its association with regular access in users of these medications.

## METHODS

A cross-sectional study was carried out with adults aged 20 years and over, who are users of the CEAF of the municipality of São Leopoldo, State of Rio Grande do Sul, Brazil, located 31.4 km from the capital, Porto Alegre. The municipality has approximately 227,000 inhabitants^d^ and a structure of public pharmaceutical services that includes two medication-dispensing units. One of them, the Central Public Pharmacy, is located in the City Center and it dispenses the medications of the CEAF, which are the subject of this study, in addition to the Basic Component of Pharmaceutical Services (CBAF) and the Strategic Component of Pharmaceutical Services (CESAF).

In 2014, the CEAF of this municipality had approximately 1,100 individuals registered, with deferred or ongoing reevaluation treatment status and with administrative referral processes, being approximately 2/3 of the services destined for the user him or herself, while the remainder were for the guardians.

In this study, we included users registered at the CEAF for at least three months, with deferred or ongoing reevaluation treatment status at the time of the interview, and those who receive medication administratively. Individuals with cognitive impairment or incapacitated to answer the questionnaire were excluded.

The sample size was calculated to contemplate the study of prevalence of adherence to the medications of the CEAF and associated factors using the program EpiInfo 7.14.0. We used the prevalence of adherence of 40%, margin of error of 5.0 percentage points, and 95% confidence level. For the study of associations, we considered statistical power of 80% with frequencies of exposure between 15% and 75%, prevalence in the non-exposed of 32%, prevalence ratio of 1.5, 95% confidence level, and addition of 10% for losses or refusals and 15% for confounding control, amounting to 412 individuals to be interviewed.

The fieldwork was developed by us. The sampling process was carried out consecutively, including users who accessed the service of the CEAF between December 12, 2014 and March 17, 2015 and those who met the inclusion criteria. After clarifying the nature of the study and signing the informed consent, the interview was conducted using a standardized questionnaire, pre-tested in a pilot study. A judicious instruction manual was used to standardize the interviews. The user was interviewed only once, even if he or she returned to the service during the investigation period. In case of refusal, the users were invited again at each return to the pharmacy during the course of the study.

The socio-demographic variables evaluated were sex (male, female), age (20–39, 40–59, 60 or more), observed race (white, non-white), marital status (married or with partner, single or without partner), education level in years (0–4, 5–8, ≥ 9), monthly family income in national minimum wages (< 2, 2–3, > 3–4, > 4), and health insurance (yes, no). As behavioral variables, we investigated smoking (non-smoker, former smoker, smoker), alcohol consumption (no consumption, less than once a week, one or more times a week), and the practice of physical activity (practice of some physical activity for at least 150 min a week, no practice^e^. As nutritional variables, we evaluated the consumption of vegetables (< 5 times a week, ≥ 5 times a week) and fruits (< 5 times a week, ≥ 5 times a week), as well as the nutritional status according to body mass index (BMI = weight, in kilograms/square of height, in meters), classified into eutrophic (BMI < 25 kg/m^2^), overweight (BMI ≥ 25 kg/m^2^ and < 30 kg/m^2^), and obese (BMI ≥ 30 kg/m^2^)^f^. To calculate BMI, we used the weight and height data reported by the users. The health characteristics investigated were self-perceived health (excellent or very good, good, fair, poor) and the number of morbidities referred by medical diagnosis (hypertension, diabetes, hypercholesterolemia, circulatory or vascular problem, osteoporosis, bronchitis or asthma, rheumatic diseases, arthritis or osteoarthritis, depression, heart problem, cancer, kidney problem, others, which were grouped into 1–2, 3–4, 5–6, and ≥ 7), in addition to the total number of medications that the user referred to use in a continuous way by medical indication, categorized into 1–3, 4–6, ≥ 7.

We also investigated the number of medications specific from the CEAF (1, ≥ 2), the type of morbidity for which they were prescribed (asthma, arthritis, transplanted organs and tissues, disorders of lipoprotein metabolism, viral hepatitis, chronic renal failure, others), and regular access in the last three months. Regular access was measured for each medication present in the monthly withdrawal receipt of the CEAF by asking: “In the past three months have you ever not get <medication> because the unit did not have it?” with the alternatives: never, once, twice or more. For those who did not get the medication at least once, we investigated how the user proceeded, asking: “And what did you do?” whose options were: did not use, used less, bought, had at home, received from donation. To measure the prevalence of access to treatment, the total number of users was used as the denominator. Regular access to CEAF medication was defined as getting all medications in the last three months, regardless of how it was obtained, by paying or from the CEAF. The alternatives of not getting (did not use, used less) and partially getting (part paid and part that did not use or used less; part received and part that did not use or used less, part that had at home and part that did not use or used less, part received from donation and part that did not use or used less) were defined as non-regular access, since the individual did not have access to all the treatment prescribed in the period investigated. Thus, regular access to the medication of the CEAF in the last three months was analyzed in three categories: no regular access, free regular access (when all medicines were provided by the Health System), and paid regular access (when all medicines were obtained out-of-pocket).

The outcome of this study was adherence to the medication of the CEAF used in the last seven days, measured by the Brief Medication Questionnaire (BMQ), in the validated version for Portuguese^2^. This instrument has three domains with questions that identify barriers to adherence in relation to regimen, beliefs, and recall regarding pharmacological treatment, classifying adherence according to a score from the number of positive responses: high adherence (no positive response), likely high adherence (1), probable low adherence (2), and low adherence (3 or more) in any domain. In the original BMQ^16^, a score of ≥ 1 was used indicating potential non-adherence in the regimen domain and positive tracing for the barriers of belief and recall. For the analysis of the data of this study, the categories probable adherence, probable low adherence, and low adherence were grouped together, being considered as adherent only the individuals that did not have barriers to adherence, that is, no positive response in the evaluated domains.

Some considerations should be made regarding the original BMQ. This instrument was developed to evaluate adherence to oral medications; however, CEAF includes medications with other pharmaceutical forms, such as injectable ones. In this study, individuals who used injectable medications during hemodialysis were considered as adherents for these medications in the regimen domain, because they received medical follow-up during this procedure. Other injectable medications dispensed by the Component, but that individuals do not receive medical follow-up during use, were classified according to the reported responses.

The data, such as medication name and dosage, used as a reference source for the judgment of the responses of users and subsequent classification of the degree of adherence by the BMQ, were those present in the monthly withdrawal receipt or in the medical prescription. This information was transcribed to the questionnaire at the beginning of the interview.

The questionnaires were reviewed and coded shortly after the interviews. Data typing was performed in the program EpiData (EpiData Association, Odense, Denmark), with double typing for error correction and automatic inconsistency checking, and the analysis was done in the statistical program Stata 11.2 (Stata Corp., College Station, United States).

The descriptive analysis characterized users according to the socio-demographic, behavioral, nutritional, anthropometric, health, and medication use variables. The questions addressed in the domains of the BMQ were analyzed individually, and the prevalence of barriers was described. The prevalence of adherence, according to each domain, was analyzed by the chi-square test for heterogeneity of proportions for the categorical variables and the chi-square test of linear trend for the ordinal variables, adopting a significance level of 5%.

To investigate the association between regular access and adherence to the medications of the CEAF, we used Poisson regression with robust variance using different analysis models. In Model 1, we evaluated the effect of the variable regular access on unadjusted adhesion. The possible confounding factors were part of the analysis of the other models. Variables associated with exposure and outcome with p ≤ 0.20 were considered as confounding factors.

This research was approved by the Research Ethics Committee of the Universidade do Vale do Rio dos Sinos (CEP 14/150). The participants signed the informed consent. Confidentiality was ensure regarding identity and information, the right not to participate, not to answer to a question, or to suspend participation at any time, ensuring the ethical aspects.

## RESULTS

We interviewed 414 users of medications of the CEAF among those eligible in the study period. Refusals amounted to less than 3%, and almost all losses because of the waiting time for the interview were recovered in subsequent months. The sample allowed us to estimate prevalence of adherence of 28%, with a margin of error of ± 4.5 percentage points, and to detect prevalence ratios of 1.49 or greater, with statistical power of 80% and 95% confidence level.

Table 1 shows that most users were female (60.9%), married or with partner (65.0%), had a mean age of 55 years (standard deviation [SD] = 13), and 7.3 years of study (SD = 4.1). Approximately 40% of the interviewees had a family income of less than two minimum wages and 63.8% had no health insurance. Half of the individuals never smoked (52.7%), 76.1% did not consume alcohol, and 77.5% were insufficiently active. Regarding the consumption of vegetables and fruits, 72% reported frequent consumption and approximately 2/3 were overweight or obese.

**Table 1:** Characteristics of users of medications of the Specialized Component of Pharmaceutical Services (CEAF). São Leopoldo, State of Rio Grande do Sul, Brazil, 2015. (n = 414)

Variable^a^	n	%
Sociodemographic		

Sex		
Male	162	39.0
Female	252	60.9
Age (years)		
20–39	59	14.3
40–59	192	46.4
≥ 60	163	39.4
Race		
White	338	81.6
Non-white	76	18.4
Marital status		
Married/With partner	269	65.0
Single/Without partner	145	35.0
Education level (years of schooling)		
0–4	107	25.9
5–8	175	42.3
≥ 9	132	31.9
Family income (monthly)^b^		
< 2 MW	160	38.7
2 to 3 MW	114	27.5
> 3 to 4 MW	58	14.0
> 4 MW	82	19.8
Health insurance		
No	264	63.8
Yes	150	36.2

Behavioral		

Smoking habit		
Non-smoker	218	52.7
Former smoker	153	37.0
Smoker	43	10.4
Alcohol consumption		
No	315	76.1
< once/week	67	16.2
≥ once/week	32	7.7
Physical activity^c^		
Active	93	22.5
Insufficiently active	321	77.5

Nutritional		

Consumption of vegetables		
< 5 times/week	111	27.7
≥ 5 times/week	290	72.3
Consumption of fruits		
< 5 times/week	109	27.2
≥ 5 times/week	292	72.8

Anthropometric		

Nutritional status		
Eutrophic	149	37.7
Overweight	156	39.5
Obesity	90	22.8

Health		

Self-perception of health		
Excellent/Very good	30	7.3
Good	147	35.5
Regular	178	43.0
Poor	59	14.3
Number of morbidities		
1–2	85	20.5
3–4	145	35.0
5–6	115	27.8
≥ 7	69	16.7

Characteristics of the use of medications		

Number of medications of continuous use		
1–3	143	34.8
4–6	147	35.8
≥ 7	121	29.4
Number of medications of the CEAF		
1	282	68.1
≥ 2	132	31.9
Regular access to the medications of the CEAF (last 3 months)		
No	106	25.7
Free access	190	46.1
Paid access	116	28.2

^a^ Maximum ignored = 21 for family income.

^b^ MW = National minimum wage in Brazil R$788.00.

^c^ Considered as active individuals who perform physical activity for at least 150 min/week.

More than half (57.3%) of the respondents evaluated their health as regular or poor, 44.5% reported more than four morbidities, and 65.2% used four or more medications of continuous use. Furthermore, 68.1% used only one medication of the CEAF and less than half (46.1%) had regular access to these medications using the Component in the last three months (Table 1).

We also observed that 414 users used 577 medications of the CEAF, most of which were antineoplastic and immunomodulatory (40.2%), represented mainly by immunosuppressants (37.1%). The ones for the respiratory system were 16.5% and those for the alimentary tract or metabolism were 9.4%, which are represented by antidiarrheals, antiinflammatory agents, intestinal antiinfectives (5.6%), and vitamins (3.8%) (data not shown in the table).

Regarding adherence to the treatment with medications of the CEAF used during the last seven days, 28.3% of the users were classified as adherent (no positive response), 38.9% as likely adherent (one positive response), 23.2% as probable low adherent (two positive responses), and 9.7% as low adherent (three or more positive responses) (data not presented in the table).

Among the questions addressed by the BMQ, we can highlight as the main barriers to adherence, in the regimen domain, the reports of failure of days or doses (8.5%) and interruption in the therapy (7.0%). In the domain that evaluated the beliefs of the individuals, 20.1% of the respondents named the medications that bothered them. In the recall domain, 52.7% received a multi-dose regimen and 16% reported having difficulty remembering to take their medications (Table 2).

**Table 2:** Frequency of positive responses to the questions addressed in the domains of the Brief Medication Questionnaire (BMQ). São Leopoldo, State of Rio Grande do Sul, Brazil, 2015. (n = 414)

Questions of the BMQ	%
Regimen Domain	16.7*
Reported interruption in therapy	7.0
Reported missing days or doses	8.5
Reduced or omitted any dose	5.1
Reported taking an extra dose or medication	3.6
Reported that he or she did not know the dosage of a medication	0.2
Refused to answer a question	0.2
Belief Domain	21.5*
Reported that some of the medications "do not work well" or "do not know"	4.6
Named the medications that bother him or her	20.1
Recall Domain	61.1*
Receives a scheme of multiple doses of medications (2 times or more/day)	52.7
Reported having difficulty remembering to take the medication	16.0

* At least one positive response in the domain.

In the evaluation of adherence according to the characteristics investigated, significantly higher prevalence of adherence was identified in single users (31.6%), with higher education (31.8%), higher income (39%), health insurance (36%), who regularly consumed vegetables (31.4%), used a single medication of the CEAF (32.3%), and had regular free access to all medications of the CEAF in the last three months (33.2%). Similar results of higher magnitude were observed in relation to the recall domain, in which higher prevalence was also observed in individuals with lower morbidity and lower number of medication of continuous use and of the CEAF. For the regimen and belief domains, regular access to the medications of the CEAF was associated with higher prevalence of adherence (p < 0.005) (Table 3).

**Table 3:** Prevalence of users adheringa to the medications of the Specialized Component of Pharmaceutical Services (CEAF) for each domain of the Brief Medication Questionnaire (BMQ), according to the characteristics investigated. São Leopoldo, State of Rio Grande do Sul, Brazil, 2015. (n = 414)

Variable	Adherence							

	BMQ		Regimen Domain		Belief Domain		Recall Domain	
			
	%	95%CI	%	95%CI	%	95%CI	%	95%CI
Total	28.3	23.9–32.6	83.3	79.7–86.9	78.5	74.5–82.5	38.9	34.2–43.6
Sex		0.066		0.280		0.348		0.535
Male	33.3	26.0–40.7	85.8	80.4–91.2	80.9	74.7–87.0	40.7	33.1–48.4
Female	25.0	19.6–30.4	81.8	76.9–86.5	77.0	71.8–82.2	37.7	31.7–43.7
Age (years)		0.923^b^		0.074^b^		0.104^b^		0.614^b^
20–39	25.4	14.0–36.9	74.6	63.1–86.0	78.0	67.1–88.9	37.3	24.6–50.0
40–59	29.7	23.2–36.2	83.9	78.6–89.1	74.0	67.7–80.2	41.7	34.6–48.7
≥ 60	27.6	20.7–34.5	85.9	80.5–91.3	84.1	78.4–89.7	36.2	28.7–43.7
Race		0.485		0.650		0.838		0.088
White	29.0	24.1–33.9	83.7	79.8–87.7	78.7	74.3–83.1	40.8	35.6–46.1
Non-white	25.0	15.0–35.0	81.6	72.7–90.5	77.6	68.0–87.2	30.3	19.7–40.8
Marital status		0.040		0.963		0.769		0.076
Married/With partner	22.1	15.2–28.9	83.5	77.3–89.6	79.3	72.6–86.0	33.1	25.4–40.9
Single/Without partner	31.6	26.0–37.2	83.3	78.8–87.8	78.1	73.1–83.0	42.0	36.1–47.9
Education level (years of schooling)		0.045^b^		0.885^b^		0.296^b^		0.000^b^
0–4	19.6	12.0–27.3	83.2	76.0–90.4	82.2	74.9–89.6	25.2	16.9–33.6
5–8	30.9	23.9–37.8	84.0	78.5–89.5	77.7	71.5–83.9	39.4	32.1–46.7
≥ 9	31.8	23.8–39.9	82.6	76.0–89.1	76.5	69.2–83.8	49.2	40.6–57.9
Family income monthly		0.050^b^		0.464^b^		0.624^b^		0.001^b^
< 2 MW	25.6	18.8–32.5	81.9	75.8–87.9	80.6	74.4–86.8	31.9	24.6–39.2
2 to 3 MW	25.4	17.3–33.6	82.5	75.4–89.5	75.4	67.4–83.5	36.8	27.9–45.8
> 3 to 4 MW	25.9	14.2–37.5	87.9	79.3–96.6	81.0	70.6–91.4	39.7	26.7–52.6
> 4 MW	39.0	28.2–49.8	84.2	76.1–92.2	76.8	67.5–86.2	54.9	43.9–65.9
Health insurance		0.008		0.784		0.662		0.000
No	23.9	18.7–29.0	83.0	78.4–87.5	79.2	74.2–84.1	30.7	25.1–36.3
Yes	36.0	28.2–43.8	84.0	78.1–90.0	77.3	70.6–84.1	53.3	45.3–61.4
Smoking habit		0.863		0.617		0.627		0.252
Non-smoker	29.4	23.3–35.5	81.7	76.5–86.8	77.1	74.8–82.7	42.7	36.0–49.3
Former smoker	26.8	19.7–33.9	85.0	79.2–90.7	81.1	74.8–87.3	34.6	27.0–42.3
Smoker	27.9	13.9–41.9	86.1	75.3–96.8	76.7	63.6–89.9	34.9	20.0–49.7
Alcohol consumption		0.270		0.597		0.408		0.040
No	27.3	22.4–32.2	84.1	80.0–88.2	78.1	73.5–82.7	35.6	30.2–40.9
< once/week	26.9	16.0–37.8	79.1	69.1–89.1	76.1	65.6–86.6	47.8	35.5–60.0
≥ once/week	40.6	22.6–58.6	84.4	71.1–97.7	87.5	75.4–99.6	53.1	34.8–71.4
Physical activity		0.737		0.269		0.391		0.314
Active	26.9	17.7–36.1	87.1	80.2–94.0	81.7	73.7–89.7	34.4	24.6–44.2
Insufficiently active	28.7	23.7–33.6	82.2	78.0–86.4	77.6	73.0–82.2	40.2	34.8–45.6
Consumption of vegetables		0.034		0.664		0.429		0.023
< 5 times/week	20.7	13.1–28.4	82.0	74.7–89.2	75.7	67.6–83.8	29.7	21.1–38.4
≥ 5 times/week	31.4	26.0–36.8	83.8	79.5–88.1	79.3	74.6–84.0	42.1	36.4–47.8
Consumption of fruits		0.212		0.949		0.924		0.509
< 5 times/week	33.0	24.1–42.0	83.5	76.4–90.6	78.0	70.1–85.9	41.3	31.9–50.7
≥ 5 times/week	26.7	21.6–31.8	83.2	78.9–87.5	78.4	73.7–83.2	37.7	32.1–43.3
Nutritional status		0.316^b^		0.937^b^		0.239^b^		0.239^b^
Eutrophic	24.8	17.8–31.9	83.2	77.2–89.3	81.2	74.9–87.6	32.9	25.3–40.5
Overweight	31.4	24.0–38.8	84.6	78.9–90.3	80.1	73.8–86.5	43.6	35.7–51.5
Obesity	30.0	20.3–39.7	83.3	75.5–91.2	74.4	65.3–83.6	38.9	28.6–49.2
Self-perception of health		0.181		0.892		0.099		0.232
Excellent/Very good	36.7	18.4–55.0	80.0	64.8–95.2	83.3	69.2–97.5	50.0	31.0–69.0
Good	32.0	24.3–39.6	85.0	79.2–90.9	83.0	76.8–89.1	42.9	34.8–51.0
Regular	27.0	20.4–33.5	82.6	77.0–88.2	77.5	71.3–83.7	36.0	28.8–43.1
Poor	18.6	8.4–28.9	83.1	73.2–92.9	67.8	55.5–80.1	32.2	19.9–44.5
Number of morbidities		0.089^b^		0.899^b^		0.152^b^		0.018^b^
1–2	31.8	21.7–41.9	82.4	74.1–90.6	80.0	71.3–88.7	45.9	35.1–56.7
3–4	31.0	23.4–38.7	83.5	77.3–89.6	81.4	75.0–87.8	40.0	31.9–48.1
5–6	27.0	18.7–35.2	85.2	78.6–91.8	78.3	70.6–85.9	40.9	31.7–50.0
≥ 7	20.3	10.6–30.0	81.2	71.7–90.6	71.0	60.0–82.0	24.6	14.2–35.1
Number of medications of continuous use		0.078^b^		0.270^b^		0.792^b^		0.037^b^
1–3	31.5	14.1–28.9	86.0	80.3–91.8	79.0	72.3–85.8	42.0	33.8–50.1
4–6	29.9	22.4–37.4	82.3	76.1–88.6	78.2	71.5–85.0	43.5	35.4–51.6
≥ 7	21.5	23.8–39.2	81.0	73.9–88.1	77.7	70.2–85.2	28.9	20.7–37.1
Number of medications of the CEAF		0.008		0.571		0.013		0.000
1	32.3	26.8–37.8	82.6	78.2–87.1	81.9	77.4–86.4	45.0	39.2–50.9
≥ 2	19.7	12.8–26.6	84.9	78.7–91.0	71.2	63.4–79.0	25.8	18.2–33.3
Regular access to CEAF medication (last 3 months)		0.049		0.000		0.002		0.028
No	19.8	12.1–27.5	69.8	60.9–78.7	79.3	71.4–87.1	33.0	23.9–42.1
Free access	33.2	26.4–39.9	91.6	87.6–95.6	71.6	65.1–78.1	45.8	38.6–52.9
Paid access	27.6	19.3–35.8	82.8	75.8–89.7	88.8	83.0–94.6	32.8	24.1–41.4

^a^ Adherents = adherence according to BMQ (no positive response).

^b^ Chi-square for linear trend.

Regarding the type of morbidity that resulted in the use of the medications of the CEAF, the most frequent were: asthma (22.3%), arthritis (14.0%), transplanted organs and tissues (12.4%), metabolic disorders of lipoproteins (9.2%), viral hepatitis (8.5%), and chronic renal failure (6.8%). Individuals with asthma and transplanted organs and tissues had the lowest prevalence of adherence (5.4% and 7.8%, respectively), while those with chronic renal failure were the most adherent to treatment (60.7%). The barriers to adherence (at least one positive response) are described in the Figure. Regarding asthma, the main barriers to adherence are in the regimen (25%) and recall (94.6%) domains. Belief was the domain that presented the greatest barrier to adherence to medication for viral hepatitis (37.1%) and arthritis (31%), whereas individuals who underwent organ transplantation reported greater barriers to adherence in the recall domain (88.2%).

**Figure:**
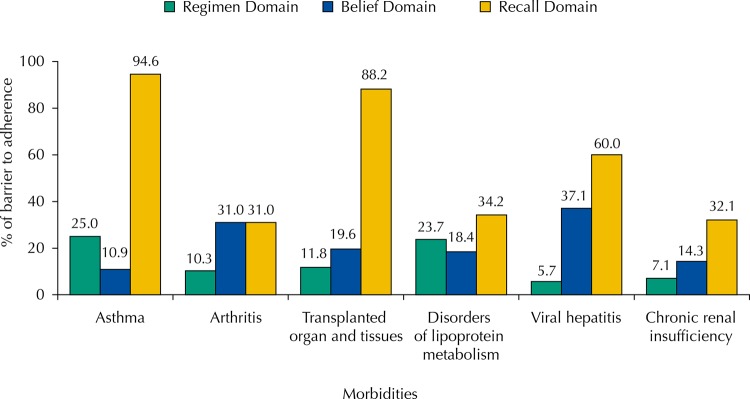
Prevalence of barriers to adherence according to the domains of the Brief Medication Questionnaire (BMQ) for the most prevalent morbidities among users of medications of the Specialized Component of Pharmaceutical Services (CEAF).

Table 4 shows the association between free regular access and adherence to the medications of the CEAF used in the last seven days, both in the crude analysis (PR = 1.67; 95%CI 1.08–2.58) and in the models adjusted for potential confounding factors. After controlling for the number of the medications of the CEAF (Model 2) and for the number of medications of continuous use (Model 3), the prevalence ratios for adherence was 1.58 (95%CI 1.03–2.44) and 1.60 (95%CI 1.04–2.48), respectively.

**Table 4:** Association between adherence to treatment and regular access to the medications of the Specialized Component of Pharmaceutical Services (CEAF) according to the various adjustment models.

Variable*	Model 1	Model 2	Model 3
	PR (95%CI)	PR (95%CI)	PR (95%CI)
Regular access to medications of the CEAF (last 3 months)			
No	1.00	1.00	1.00
Free access	1.67 (1.08–2.58)	1.58 (1.03–2.44)	1.60 (1.04–2.48)
Paid access	1.39 (0.86–2.26)	1.20 (0.74–1.95)	1.32 (0.81–2.13)
Number of medications of the CEAF			
1		1.61 (1.10–2.37)	
≥ 2		1.00	
Number of medications of continuous use			
1–3			1.49 (0.98–2.27)
4–6			1.39 (0.91–2.13)
≥ 7			1.00

Model 1: unadjusted access effect; Model 2: access adjusted by the number of medications of the CEAF; Model 3: access adjusted by the number of medications of continuous use.

* Variables associated with outcome and exposure at the significance level p ≤ 0.2.

## DISCUSSION

The study found low prevalence of adherence to medications of the CEAF by the users, being it proportionally lower in the recall domain. Regular access was associated with adherence to medications used in the last seven days, even after adjusting for number of medications, with users being 60% more likely to adhere to treatment if they received the drug free of charge from the CEAF in the three months prior to the interview.

The low prevalence of adherence to the pharmacological therapy identified in this study is consistent with the literature, which indicates that 28.1% to 88.2%^1^ ^,^ ^4^ ^,^ ^6^ ^,^ ^10^ of users adhere to treatment. However, this comparability should be made carefully, since the studies mentioned have used different methodologies, populations, and morbidities, as well as different instruments for the evaluation and categorization of the outcome, which may explain the large range in the prevalence of adherence. The result found can be explained by the high proportion of individuals who reported recall barriers, which has also been found in hypertensive patients using the same instrument^2^ ^,^ ^16^ ^,^ ^17^. In this study, hypertensive individuals corresponded to 54.0% of the sample, justifying, in part, the similarity in the findings. Another relevant aspect is the complexity of the treatment, which can be a limiting factor for adherence. As an example, we highlight the pharmacological therapy indicated for transplanted users, 88% of whom presented barriers in this domain. However, simplifying the regimen does not only mean reducing the number of medications or the frequency of daily doses, but it also requires a shared management of the care between the health professional and the user, with the adoption of strategies aimed at the individual needs that promote the rational use of medication^14^ ^,^ ^16^.

In relation to the other domains, belief and regimen, the prevalence of barriers found are in accordance with the original validation study of the BMQ^16^, in which the belief domain was the second most prevalent for barriers; however, it presented an inverse order when compared to other Brazilian studies performed with hypertensive patients^2^ ^,^ ^17^. These differences in relation to this study can be explained by the proportion of individuals who regularly undergo hemodialysis, when the medication of the CEAF is applied. Thus, these individuals tend to adhere more in this domain because of the need to perform hemodialysis, considering that non-adherence is more likely to occur in situations when the users administer themselves the medication. We highlight that users with chronic renal failure were those with the highest prevalence of adherence.

Analyzing the factors associated with adherence, we observed that, in the crude analysis, individual characteristics such as better socioeconomic conditions and better education level were factors associated with adherence; however, only the characteristics of drug use remained associated after adjustment. The results found are in line with other Brazilian studies^14^ ^,^ ^15^ ^,^ ^17^.

In this study, users who regularly received medications from the CEAF presented a 60% higher probability of adherence in the last week when compared to users whose access to the medication or pharmacological treatment was interrupted or underutilized in the period of three months. The association between regular access and adherence to the medications of the CEAF has not been evaluated in the available pharmacoepidemiological studies. However, the prevalence of free access found was similar to the findings of Boing et al.^3^, who have identified a prevalence of 45.3% of access to all medications from the SUS when prescribed in the system itself. It is well known that access to medication is a major obstacle to adherence, and free availability, by the public system, is the main current barrier faced by users. In countries such as Brazil, where there are large numbers of low-income families, this factor becomes an aggravating factor for the continuity of the pharmacological treatment^9^. In this study, the recall period used for adherence was the last week, and for access, the last three months. Thus, it is plausible to think that regularity in the free access to the medications of the CEAF has an impact on the behavior of users, contributing to their commitment with treatment and self-care. In addition, for individuals with paid regular access, no association was found between access and adherence.

Because of the cross-sectional design, the association between regular access and adherence to pharmacological therapy, even when using distinct recall periods, may have been affected by reverse causality, since exposure and outcome were measured at the same time. Thus, longitudinal studies are important to elucidate the associations described herein. Another limitation of the study refers to the use of self-report to measure adherence to pharmacological treatment, being subject to recall bias. Furthermore, the sample of this study consists of users of the CEAF who get their medication, allowing the generalization of the results for this population. We highlight that the users of medications of the CEAF have a lower average age, higher education level, and use more medications daily when compared to the users of the Basic Component of Pharmaceutical Services^11^.

Despite these limitations, this is the first study to evaluate adherence to the medications of the CEAF and associated factors, as well as the regularity of access in a three-month period. The results found reinforce the low prevalence of adherence to pharmacological therapy and they identify its relation with free regular access. By knowing the monthly amount of each medication to supply the treatment of users of this Component, it is surprising that more than half face irregular access, evidencing a significant limitation of the drug policies in Brazil. The creation of the CEAF was motivated by the need to expand free access to high-cost medications^g^; however, in the case of users with lower purchasing power, the lack of regularity in the access may compromise family income^13^, lead to underutilization of the medication, or even lead to a total interruption of the use^12^.

It is a fact that public investments in health, especially those related to high-cost medications, have been growing in recent years^18^; however, in parallel, there is increasing demand, hindering availability to all users. Thus, an adequate CEAF programming is essential, as well as an effective pharmacotherapeutic follow-up of the users by the health teams to promote adherence to treatment.

## Footnotes

a World Health Organization. Adherence to long-term therapies: evidence for action. Geneva; 2003 [cited 2017 Jul 19]. Available from: http://www.who.int/chp/knowledge/publications/adherence_report/en/

b Ministério da Saúde (BR). Portaria nº 2981, de 26 de novembro de 2009. Aprova o componente Especializado da Assistência Farmacêutica. Brasília (DF); 2009 [cited 2017 Jul 19]. Available from: http://www1.saude.rs.gov.br/dados/1332344988133Portaria%20GM%20MS%20n.%202981%20de%2026-11-2009.pdf

c Ministério da Saúde (BR). Portaria nº 204, de 29 de janeiro de 2007. Regulamenta o financiamento e a transferência dos recursos federais para as ações e serviços de saúde, na forma de blocos de financiamento, com o respectivo monitoramento e controle. Brasília (DF); 2007. Available from: http://bvsms.saude.gov.br/bvs/saudelegis/gm/2007/prt0204_29_01_2007_comp.html

d Instituto Brasileiro de Geografia e Estatística. Cidades@ : São Leopoldo-RS. Rio de Janeiro: IBGE; 2014 [cited 2015 Jun 16]. Available from: http://www.cidades.ibge.gov.br/xtras/perfil.php?lang=&codmun=431870&search=rio-grande-do-sul|sao-leopoldo

e World Health Organization. Global recomendations on physical activity for health Geneva; 2010 [cited 2017 Jul 24]. Available from:http://apps.who.int/iris/bitstream/10665/44399/1/9789241599979_eng.pdf

f World Health Organization. Diet, nutrition, and the prevention of chronic diseases: report of a Joint WHO/FAO Expert Consultation. Geneva; 2003 [cited 2017 Jul 24]. (WHO Technical Report Series, 916). Available from: http://apps.who.int/iris/bitstream/10665/42665/1/WHO_TRS_916.pdf

g Ministério da Saúde, Secretaria de Ciência, Tecnologia e Insumos Estratégicos, Departamento de Assistência Famacêutica, Insumos Estratégicos. Da excepcionalidade às linhas de cuidado: o Componente Especializado da Assistência Farmacêutica. Brasília(DF); 2010 [cited 2017 Jul 19]. (Série B. Textos Básicos de Saúde). Available from: http://bvsms.saude.gov.br/bvs/publicacoes/excepcionalidade_linhas_cuidado_ceaf.pdf
